# Metric discordance under MIDOG++ domain shift: a leave-one-domain-out audit of F1, emitted-detection confidence calibration, and ROI count error

**DOI:** 10.1016/j.jpi.2026.100705

**Published:** 2026-07-31

**Authors:** Cleverson de Souza

**Affiliations:** Department of Comparative, Diagnostic & Population Medicine, University of Florida College of Veterinary Medicine, Gainesville, FL, USA

**Keywords:** Mitotic figures, Calibration, Domain shift, Computational pathology, MIDOG, Model reliability, Comparative pathology

## Abstract

**Background:**

Mitotic-figure detectors are usually evaluated by detection accuracy, but use also depends on confidence and count reliability under domain shift.

**Methods:**

We evaluated MIDOG++ with leave-one-domain-out testing across seven domains. Adaptive-bin detection expected calibration error (D-ECE1) measured the bin-mass-weighted mean absolute confidence-precision gap among post-non-maximum suppression detections retained at a 0.05 score floor; centroid-distance matching supplied the true positive/false positive labels. F1 and count error were evaluated at the fixed 0.5 operating threshold. Baseline and domain-adversarial RetinaNet arms were compared as a controlled perturbation, with exploratory domain-composition resampling and two secondary seeds.

**Results:**

No directionally stable aggregate calibration advantage was established across three seed series. In the primary series, macro D-ECE1 was 0.0786 for baseline and 0.0741 for the domain-adversarial arm (paired difference, −0.0045), but two additional seed series produced paired differences of +0.0287 and −0.0068. A provenance audit confirmed the secondary series were generated under the locked protocol; they were reported as a sensitivity comparison and were not pooled with the primary point estimate. The fixed-domain case-bootstrap interval was below zero, whereas exploratory domain-composition resampling crossed zero (−0.0213 to +0.0112). The two domains with F1 gains had worse D-ECE1. Lymphoma/lymphosarcoma had the highest observed D-ECE1 in both arms and was systematically undercounted.

**Conclusions:**

In this benchmark, F1, emitted-detection calibration, count behavior, and maximum observed-domain D-ECE1 were not interchangeable endpoints. The tested domain-adversarial configuration did not establish a directionally stable calibration benefit. Evaluations should report detection-specific calibration, retained-detection denominators, count consequences, and finite-benchmark observed-domain D-ECE1 alongside F1.

## Highlights

• MIDOG++ leave-one-domain-out evaluation compared accuracy, calibration, and counts.
• F1 gains could coincide with worse emitted-detection calibration.
• Domain-adversarial training served as a controlled stress test.
• Lymphoma/lymphosarcoma showed systematic undercounting in both model arms.
• Species regroupings were descriptive because species is confounded.

## Introduction

Mitotic-figure detection is a natural target for computational pathology because the object is discrete, countable, and clinically consequential. In human and veterinary tumors, mitotic count contributes to grading and prognosis.^1^ The MIDOG and MIDOG++ challenges have therefore become important reference benchmarks for domain generalization in histopathology, showing that mitotic detectors can lose performance when tumor type, scanner, lab context, or species-associated image distributions change.^1^, ^2^, ^3^

Challenge-style evaluations of mitotic-figure detectors commonly emphasize detection accuracy, usually summarized by F1 or related operating-point metrics. The MIDOG/MIDOG++ challenge literature uses F1 as a principal operating-point summary and also reports precision, recall, and average precision (AP).^1^, ^2^, ^3^ These are appropriate detection endpoints, but they do not answer whether emitted confidence scores track precision, how retained detections change the count burden, or whether calibration error is concentrated in a particular held-out domain. Accuracy is necessary, but it is not the only reliability property a detector may need. Users may interpret a detector score of 0.90 as indicating high confidence in correctness, although raw detector scores are not automatically calibrated probabilities.

The insufficiency claim in this study is therefore specific rather than absolute: F1, precision, recall, and AP remain valid measures of detection performance, but they are incomplete for a pathology-facing reliability assessment. The present benchmark tests whether those conventional endpoints can diverge from matching-conditioned confidence calibration, operating-point count consequences, and the maximum observed-domain burden. It does not assume that calibration replaces accuracy or that a single calibration score is a universal clinical decision rule.

Calibration is therefore a distinct endpoint from accuracy. Modern neural networks can be miscalibrated even when accurate,^4^ and object detectors require calibration metrics that respect localization and precision rather than classification accuracy alone.^5^, ^6^ For mitotic-figure detection, calibration can be conditioned on the same one-to-one matching rule used for accuracy so that each emitted confidence receives a reproducible true positive (TP)/false positive (FP) label. In this formulation, matching supplies the label; localization is not a second calibrated variable.

Average calibration is also insufficient. A detector can appear reasonably calibrated on a pooled or macro-average summary while retaining a high one-dimensional detection expected calibration error (D-ECE1) in one held-out domain. That failure mode matters because a single tumor type, scanner context, or lab workflow may become the dominant use case. Medical artificial intelligence (AI) fairness work has warned that subgroup failures can be hidden by pooled summaries,^7^ and worst-group approaches formalize the idea that average performance is not enough under group shift.^8^ We therefore report the maximum observed-domain D-ECE1 as a finite-benchmark disparity summary, not as a general future-domain guarantee.

MIDOG++ creates an especially useful setting for this question because it contains multiple human and canine tumor domains, multiple scanner contexts, and substantial domain shift. It also creates a comparative-pathology temptation that must be handled carefully. The species axis is not independently identifiable because human and canine specimens do not share tumor types, and scanner context is strongly entangled with species. A human-versus-canine contrast in this dataset is therefore a descriptive regrouping, not an estimate of a causal species effect.

In this study, we use MIDOG++ as a leave-one-domain-out (LODO) audit. The primary goal is not to produce a new leaderboard detector, but to test whether commonly reported detection accuracy captures the reliability properties needed for pathology-facing use. We evaluate emitted-detection calibration with D-ECE1, quantify the maximum observed-domain D-ECE1 as a finite-benchmark disparity summary, and compare these quantities with operating-point F1 and case-level count consequences.

As a controlled stress test, we compare a no-adaptation RetinaNet with an otherwise matched domain-adversarial RetinaNet. Domain-adversarial training is a plausible intervention because it attempts to reduce source-domain predictability in learned features.^9^ In computational pathology, domain-generalization methods are usually judged by accuracy.^10^ In MIDOG++, however, source-domain labels are composite: they include technical context such as scanner and lab, but also tumor type, tissue architecture, mitotic burden, and species-associated morphology. For that reason, the domain-adversarial arm is best interpreted as a controlled perturbation against composite source-domain identity, not as clean isolation of scanner or lab invariance.

The study inherits the MIDOG++ specimens, organizer domains, LODO framing, official matching context, and RetinaNet technical-validation lineage.^1^, ^2^ What is new here is the reliability audit around that benchmark: adaptive-bin matching-conditioned confidence calibration, retained-detection denominators, burden-normalized count consequences, seed/provenance sensitivity, and the maximum observed-domain summary. RetinaNet was retained for continuity with the established MIDOG++ reference approaches and to hold architecture and detector family fixed while testing the domain-adversarial perturbation. Newer YOLO-family, transformer-based, and segmentation-based detectors may be important comparators, but they are outside the scope of this controlled two-arm benchmark.

This study makes three contributions. First, it evaluates mitotic-figure detectors with a matching-conditioned confidence calibration metric whose TP/FP labels use the same assignment rule as accuracy. Second, it reports the maximum observed-domain calibration burden as a finite-benchmark disparity summary for MIDOG++ LODO evaluation. Third, it shows that accuracy, calibration, and count error can move in different directions under domain shift. The species-axis analysis is retained as a brief design limitation rather than as a primary contribution.

## Materials and methods

### Study design

This was a controlled methodological benchmark on a public dataset. No human or animal subjects were enrolled for this study. The final run configuration and core endpoints were fixed before the final comparative analysis and recorded in a run manifest. The primary reliability endpoint was macro out-of-domain D-ECE1 across the seven LODO folds. The primary disparity endpoint was the maximum observed-domain D-ECE1. The model comparison was domain-adversarial RetinaNet versus a no-adaptation RetinaNet baseline trained and evaluated under the same split, sampling, inference, matching, and calibration rules.

The study was designed to test a domain-adversarial training configuration rather than to maximize detection performance. Both model arms used the same architecture family, training duration, patch size, sampling policy, matching oracle, non-maximum suppression (NMS) setting, confidence floor, and operating-point threshold. The held-out domain was never used in training, model selection, or recalibration. Because MIDOG++ organizer domains are composite labels, the adversarial target mixed tumor type, species, scanner, lab context, tissue architecture, and mitotic burden rather than isolating a single technical nuisance factor.

### Dataset

We used MIDOG++, a public multi-domain mitotic-figure dataset, with 503 specimens from seven tumor domains and 11,937 non-deleted mitotic figures (Table 1).^1^ The MIDOG++ data descriptor reports 14,351 non-mitotic-figure (imposter) annotations^1^; the released MIDOG++ database we analyzed contains 14,349 imposter point annotations, all non-deleted. The analysis used these 14,349. Imposter-anchored patches constituted 25% of sampled patches. Each specimen contributed one region of interest (ROI), so the specimen and image were the same splitting unit in this benchmark. The seven organizer domains were three human domains, breast carcinoma, neuroendocrine tumor, and melanoma, and four canine domains, lung carcinoma, lymphoma/lymphosarcoma, soft-tissue sarcoma, and cutaneous mast cell tumor. The source metadata used by this analysis labels this domain as canine lymphoma; the MIDOG++ data descriptor names the same 55-specimen 3DHistech domain lymphosarcoma. The source dataset describes expert ROI selection within tumor areas with appropriate tissue and scan quality and high mitotic density, and exclusion of cases with particularly poor tissue or scan quality throughout the whole-slide image.^1^ This analysis did not add a separate image-quality score or post hoc slide-quality exclusion beyond that dataset/source selection.

**Table 1:** MIDOG++ organizer-domain composition used for leave-one-domain-out evaluation.

Domain	Species	Scanner context	Specimens	Mitotic figures
Breast carcinoma	Human	Hamamatsu XR/S360; Aperio CS2	150	1721
Neuroendocrine tumor	Human	Hamamatsu	55	639
Melanoma	Human	Hamamatsu	49	1150
Lung carcinoma	Canine	3DHistech	44	855
Lymphoma/lymphosarcoma	Canine	3DHistech	55	3959
Soft-tissue sarcoma	Canine	3DHistech	100	1286
Cutaneous mast cell tumor	Canine	Aperio	50	2327
Total			503	11,937

### Matching rule and coordinate handling

All accuracy and calibration quantities used the same detection-to-ground-truth assignment. True-positive and false-positive labels were assigned with the official MIDOG centroid-distance matching rule, vendored from evalutils 0.3.1. Each emitted detection could match at most one ground-truth point within the challenge tolerance radius, and unmatched ground-truth points were false negatives (FNs) for operating-point F1. This makes calibration a matching-conditioned confidence functional: the assignment determines the TP/FP label, but localization is not itself a separately calibrated dimension.

The matcher operates in physical units, so detections produced in pixel coordinates were converted using each image's microns-per-pixel metadata. The pipeline checked that resolution metadata resolved for every image rather than silently falling back to unit scale. This check was necessary because an incorrect pixel-to-micrometer conversion would change the effective matching radius and could distort both F1 and calibration labels.

### Detection calibration

The primary calibration metric was detection expected calibration error, D-ECE1, in the precision-conditioned form used for object detection.^5^, ^6^ In this one-dimensional implementation, the numeral 1 denotes one calibration dimension; it should not be read as an L1-norm label. The scalar discrepancy used here is the bin-mass-weighted mean absolute confidence-precision gap. Post-NMS detections with confidence at least 0.05 were sorted by confidence and partitioned into 10 adaptive equal-frequency bins. Within each occupied bin, mean confidence was compared with observed precision, defined as the fraction of emitted detections in that bin that were TPs under the matching rule. Tied scores could collapse nominal quantile bins, and the effective number of occupied bins was recorded. We refer to this implementation as adaptive-bin D-ECE1. It is conditioned on retained emitted detections and therefore does not measure missed mitoses directly; recall, F1, and count error were evaluated separately.

Equal-frequency bins were used because detector confidence distributions can be skewed; equal-width bins can leave some confidence regions sparsely populated.^11^, ^12^ Reliability diagrams were computed as descriptive complements; adaptive-bin D-ECE1 remained the primary calibration endpoint. The calibration score floor of 0.05 was deliberately separate from the fixed F1 operating threshold so that calibration was evaluated across the retained confidence range rather than only near the decision threshold. Because the two arms can emit different numbers and distributions of retained detections, the arm comparison estimates an end-to-end emitted-detection reliability functional rather than a recalibration effect holding a common candidate set fixed.

### Models and training

The detector was a RetinaNet with a ResNet-50 feature-pyramid backbone and focal loss.^1^, ^2^, ^13^ We selected this family for continuity with the MIDOG++ technical-validation/reference lineage and to provide a controlled one-stage focal-loss detector in which the training objective, rather than the detector family, was perturbed. The baseline arm used no domain-adversarial training. The domain-adversarial arm added a gradient-reversal domain head following Ganin et al.^9^ The domain head operated on pooled feature-pyramid features and predicted which of the six source domains a training patch came from. The held-out domain was not used by the domain classifier, so this was a domain-generalization use of the gradient-reversal idea rather than transductive adaptation to a target domain. Because the source-domain label was composite, the experiment tested adversarial training against composite source-domain identity, not isolated scanner or lab nuisance invariance. The reversal weight followed the standard schedule from 0 to 1 over training. The saved logs provide a limited branch-activity check through the final domain-head loss and reversal weight, but no validation domain accuracy or frozen-feature probe; therefore, they do not establish domain-invariant representations.

Both arms were trained for 20 epochs with batch size 16, learning rate 5e-5, AdamW optimization, automatic mixed precision, 512 by 512 pixel patches, 12 patches per specimen per epoch, and fixed random seed 0 for the locked primary comparison. Source-domain sampling was balanced in both arms. Imposter annotations were used through hard-negative-aware background sampling, with a hard-negative fraction of 0.25, rather than as a second foreground class. This choice was held constant across arms but can influence FP prevalence and confidence calibration in domains with high imposter density. Mitotic-figure point annotations were converted to fixed boxes centered on the annotated point to provide RetinaNet regression targets.

### Leave-one-domain-out protocol

The evaluation protocol was LODO. For each fold, a model was trained on six domains and evaluated on the held-out seventh. This was repeated for each of the seven domains and for both arms, yielding 14 final fold outputs. Each held-out ROI was processed by overlapping 512 by 512 pixel sliding windows with 20% overlap. Window detections were merged by global NMS at intersection-over-union 0.4. Operating-point F1 was computed at confidence threshold 0.5 with the same matching rule used to label detections for calibration. The 0.5 value was a fixed protocol operating point, not a domain-specific or clinically optimized threshold.

No post-hoc recalibration was applied. This was intentional: the primary endpoint was raw out-of-domain reliability, the behavior a user would encounter when the detector is applied to an unseen domain without target-domain fitting.

### Statistical analysis

Per-domain D-ECE1 uncertainty was estimated by case-level cluster bootstrap with 2000 replicates. Cases within the held-out domain were resampled with replacement, and the full D-ECE1 calculation, including equal-frequency bin edges, was recomputed in each replicate. Within a domain, the point estimate is detection-weighted because cases with more retained detections contribute more; case resampling preserves dependence but does not make the point estimate case-balanced. The case was the resampling unit because detections cluster within specimens; treating detections as independent would understate uncertainty, especially in domains with many mitotic figures per case.

The primary reliability summary was the equal-weight macro mean of D-ECE1 across the seven held-out domains. The primary disparity summary was the maximum observed-domain D-ECE1. The bootstrap recomputed the maximum across domains inside each replicate; the resulting raw bootstrap-maximum distribution was used to describe conditional finite-benchmark uncertainty. We did not treat an ad hoc bias-corrected maximum as a separate primary point estimate. Dispersion was summarized by the max-min spread and cross-domain standard deviation.

The model contrast was summarized as paired domain-level Delta D-ECE1, defined as D-ECE1 for domain-adversarial training minus D-ECE1 for baseline in the same held-out domain. Negative values indicate better calibration under the domain-adversarial arm. Paired case-bootstrap intervals for each domain resampled specimens within the held-out domain and recomputed both arm-specific D-ECE1 values on the same resampled specimen set. We report a fixed-domain interval for the mean paired delta and an exploratory domain-composition resampling sensitivity that resampled the seven observed domains with replacement before resampling cases within selected domains. The latter was not interpreted as an inferential interval for arbitrary future domains. Because only seven domain pairs are available, the paired tests were treated as descriptive support rather than as decisive inference. The report includes the Wilcoxon signed-rank test and an exact sign test. We emphasize intervals and direction pattern over thresholded significance testing.

After the locked primary analysis, the same 14-fold training and evaluation grid was repeated with two additional fixed training seeds, 1 and 2, as a secondary sensitivity analysis. These secondary outputs were summarized separately from the primary seed-0 final-protocol analysis and were not pooled with the primary estimates. A post-hoc provenance audit established their equivalence to the locked protocol. All 28 secondary runs completed the full 20-epoch schedule; no non-finite optimizer step occurred in any run, so the max_nonfinite_steps safeguard was never triggered; this field also takes both recorded values (null and 20) within the verified primary series and is therefore not protocol-defining. The training configuration matched the corresponding primary run on every field except the intended seed, and the macro Delta D-ECE1 values reproduced exactly through the locked analysis path, with the primary seed-0 delta recomputed as an in-audit control. The archived NPZ files retain a conservative unverified-or-pilot provenance tag applied at generation time; the audit result, not the tag, governs their interpretation, and the secondary series was reported separately from the primary point estimate. The seed-sensitivity endpoint was qualitative robustness of the macro Delta D-ECE1 direction and maximum observed-domain calibration behavior.

Case-level count consequences were summarized at the same fixed F1 operating threshold when slide-level counts were available. True count was TP + FN, predicted count was TP + FP, count bias was predicted minus true count, and count mean absolute error was summarized by held-out domain and arm with case-level bootstrap intervals. We also report mean true and predicted counts, the predicted/true count ratio, signed relative bias, and case-normalized absolute count error. The latter is a descriptive sensitivity whose denominator is the case true count with a floor of 1 for zero-count cases; it is not treated as a definitive burden-independent endpoint. Threshold-sweep sensitivity was not reported because the locked NPZ files contain retained prediction coordinates and scores but not the corresponding ground-truth coordinates required to re-run the one-to-one matching procedure at new score floors. All calibration conclusions therefore remain restricted to detections retained at the prespecified 0.05 score floor. Grade-threshold crossing risk was not reported because no tumor-specific mitotic-count cutoff had been locked and citation-verified for this output.

### Species-axis identifiability analysis

The species axis was treated as a structural limitation. No tumor type appears in both human and canine specimens, and the scanner is almost completely entangled with species. A human-versus-canine difference in MIDOG++ is therefore a mixture of species, tumor type, scanner, and lab context. We retain the human/canine regrouping only as a confounded descriptive contrast and make no causal species claim; it is not a primary endpoint.

### Reproducibility and quality control

The final primary run used protocol version midog-lodo-audit-2026-05-30-v1. The analysis failed closed unless all 14 expected current-protocol fold outputs were present. The final analyze and report jobs consumed exactly the 14 top-level LODO NPZ files and no archived or quarantined outputs. Regression tests covered the matching oracle, calibration metrics, report count metrics, threshold-reporting guardrails, training-protocol invariants, and gradient-reversal behavior. The final analysis completed without recorded execution errors. An independent numerical check recomputed all 14 D-ECE1 point estimates, F1 values, count point estimates, macro summaries, and paired deltas directly from the NPZ files and matched the JSON outputs; bootstrap intervals were checked for point-estimate bracketing but were not regenerated in that independent check. Secondary seed-sensitivity runs for seeds 1 and 2 completed all 28 expected fold outputs and were summarized separately; a post-hoc audit confirmed they completed the full training schedule under a configuration identical to the primary runs except for the seed, and their macro Delta D-ECE1 values reproduced exactly through the locked analysis path. Saved training logs were also summarized for final domain-head cross-entropy and gradient-reversal schedule state, but they did not contain domain-head accuracy or a frozen-feature domain probe.

## Results

### Final fold set and data integrity

The final analysis included all 14 planned current-protocol fold outputs: seven held-out MIDOG++ domains for the baseline model and seven matched held-out domains for the domain-adversarial model. Each fold had aligned detection-confidence, TP-label, and case-identifier arrays, finite scores in the expected range, current protocol metadata, and operating-point TP, FP, and FN counts. The held-out specimen counts were 150 for breast carcinoma, 55 for neuroendocrine tumor, 49 for melanoma, 44 for lung carcinoma, 55 for lymphoma/lymphosarcoma, 100 for soft-tissue sarcoma, and 50 for cutaneous mast cell tumor. No archived, pre-audit, quarantined, or partial fold outputs were included.

### Per-domain accuracy and detection calibration

Per-domain F1 and D-ECE1 varied substantially across held-out domains (Table 2). Baseline F1 ranged from 0.531 in canine lung carcinoma to 0.794 in human melanoma. Domain-adversarial F1 ranged from 0.501 in canine lymphoma/lymphosarcoma to 0.746 in human melanoma. Macro F1 was 0.6598 for baseline and 0.6488 for domain-adversarial training.

**Table 2:** Per-domain F1 and detection calibration error.

Domain	Arm	F1	D-ECE1	95% CI
Breast carcinoma	Baseline	0.690	0.037	0.033–0.050
Breast carcinoma	Domain-adversarial	0.642	0.037	0.033–0.043
Neuroendocrine tumor	Baseline	0.566	0.059	0.057–0.074
Neuroendocrine tumor	Domain-adversarial	0.633	0.067	0.059–0.076
Melanoma	Baseline	0.794	0.064	0.056–0.075
Melanoma	Domain-adversarial	0.746	0.049	0.043–0.063
Lung carcinoma	Baseline	0.531	0.044	0.039–0.056
Lung carcinoma	Domain-adversarial	0.596	0.081	0.074–0.086
Lymphoma/lymphosarcoma	Baseline	0.555	0.180	0.149–0.213
Lymphoma/lymphosarcoma	Domain-adversarial	0.501	0.182	0.153–0.210
Soft-tissue sarcoma	Baseline	0.772	0.082	0.077–0.087
Soft-tissue sarcoma	Domain-adversarial	0.739	0.050	0.039–0.058
Cutaneous mast cell tumor	Baseline	0.710	0.083	0.069–0.101
Cutaneous mast cell tumor	Domain-adversarial	0.685	0.052	0.045–0.068

**Fig. 1:**
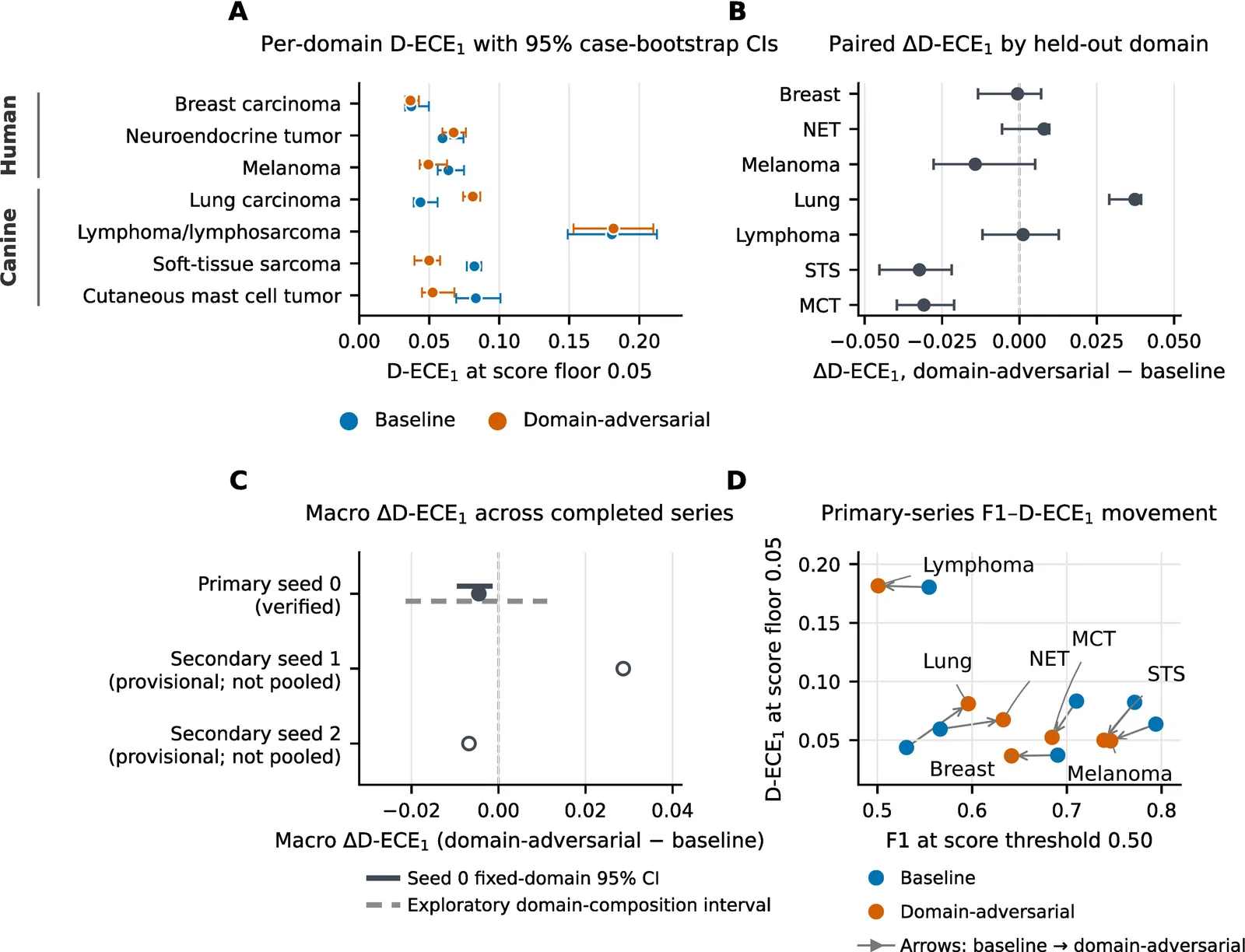
Primary-series calibration and endpoint discordance under leave-one-domain-out shift, with secondary-series sensitivity. (A) Per-domain adaptive-bin D-ECE1 among post-NMS detections retained at a score floor of 0.05; points indicate arm-specific estimates and horizontal bars indicate 95% case-bootstrap confidence intervals. (B) Paired domain-level ΔD-ECE1, calculated as domain-adversarial minus baseline; negative values indicate lower D-ECE1 in the domain-adversarial arm. Horizontal bars indicate 95% paired case-bootstrap confidence intervals. (C) Macro ΔD-ECE1 across the three completed seed series. Seed 0 was the primary series; seeds 1 and 2 were provenance-audited sensitivity series and were not pooled with the primary point estimate. The solid interval represents the seed-0 fixed-domain 95% case-bootstrap CI, whereas the dashed interval represents an exploratory domain-composition resampling sensitivity to the composition and weighting of the seven observed domains, not an interval for arbitrary future domains. (D) Primary-series movement in F1 at score threshold 0.50 and D-ECE1 at score floor 0.05; labels use breast, NET (neuroendocrine tumor), melanoma, lung, lymphoma/lymphosarcoma, STS (soft-tissue sarcoma), and MCT (cutaneous mast cell tumor); arrows point from baseline to domain-adversarial training.

**Fig. 2:**
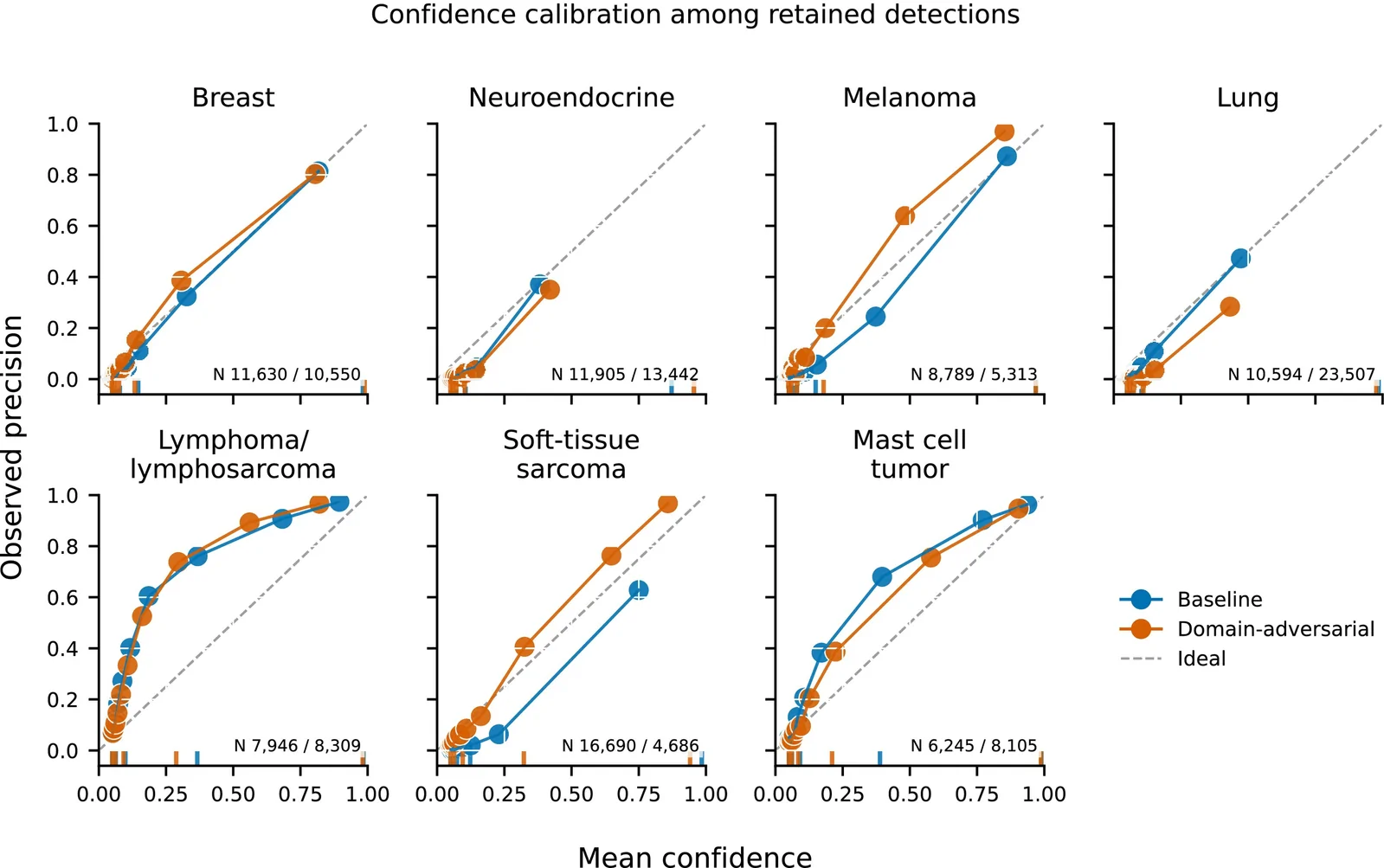
Reliability diagrams by held-out domain. Mean confidence versus observed precision in adaptive equal-frequency bins for each held-out domain and model arm. Detections below the score floor of 0.05 were excluded before binning, and tied scores could collapse nominal quantile bins. Retained-detection N is shown for each domain/arm, and compact retained-score quantile rugs expose the score range in each arm. Marker area reflects bin occupancy. The diagonal line indicates ideal calibration.

Calibration did not track accuracy in a simple way. Baseline D-ECE1 ranged from 0.037 in human breast carcinoma to 0.180 in canine lymphoma/lymphosarcoma. Domain-adversarial D-ECE1 ranged from 0.037 in human breast carcinoma to 0.182 in canine lymphoma/lymphosarcoma. Canine lymphoma/lymphosarcoma had the highest observed D-ECE1 in both arms despite not being the lowest-F1 domain in the baseline arm (Fig. 1A, B). The per-domain reliability diagrams underlying these calibration errors are shown in Fig. 2.

At the locked 0.05 score floor, the primary macro D-ECE1 delta was −0.0059 with 5 bins, −0.0044 with 8 bins, −0.0045 with 10 bins, −0.0065 with 15 bins, −0.0060 with 20 bins, −0.0058 with 30 bins, and −0.0052 with 50 bins. This bin-count sensitivity did not change the sign of the seed-0 macro contrast, but it did not test a different score floor or re-run matching. The retained-detection denominators and per-arm confidence-label summaries that underlie these calibration estimates are reported in Table 3.

**Table 3:** Retained-detection denominators and confidence-label summaries at the 0.05 score floor. Mean confidence and empirical TP fraction are calculated over retained emitted detections; case-level *N* is the median number of retained detections per case with the interquartile range in parentheses.

Domain	Arm	Retained *N*	Case-level *N*, median (IQR)	Mean confidence	Empirical TP fraction
Breast carcinoma	Baseline	11,630	62.0 (34.25–105.75)	0.1788	0.1415
Breast carcinoma	Domain-adversarial	10,550	53.0 (32.25–89.00)	0.1721	0.1541
Neuroendocrine tumor	Baseline	11,905	176.0 (110.50–284.50)	0.1088	0.0493
Neuroendocrine tumor	Domain-adversarial	13,442	204.0 (138.50–311.00)	0.1119	0.0445
Melanoma	Baseline	8789	159.0 (88.00–203.00)	0.1889	0.1274
Melanoma	Domain-adversarial	5313	93.0 (42.00–131.00)	0.2020	0.2102
Lung carcinoma	Baseline	10,594	206.0 (143.25–309.00)	0.1177	0.0739
Lung carcinoma	Domain-adversarial	23,507	492.5 (335.25–672.25)	0.1167	0.0356
Lymphoma/lymphosarcoma	Baseline	7946	135.0 (91.00–191.00)	0.2579	0.4383
Lymphoma/lymphosarcoma	Domain-adversarial	8309	138.0 (101.00–195.50)	0.2266	0.4082
Soft-tissue sarcoma	Baseline	16,690	140.0 (79.50–226.50)	0.1575	0.0753
Soft-tissue sarcoma	Domain-adversarial	4686	34.0 (20.75–56.25)	0.2433	0.2544
Cutaneous mast cell tumor	Baseline	6245	68.5 (37.25–155.50)	0.2704	0.3473
Cutaneous mast cell tumor	Domain-adversarial	8105	91.0 (54.75–220.25)	0.2243	0.2650

### Domain-adversarial training produced mixed calibration changes

The domain-level comparison gave a mean paired Delta D-ECE1 of −0.0045 (Table 4, Fig. 1B). The paired case-bootstrap interval that treated the seven observed domains as fixed was −0.0096 to −0.0014, consistent with a small average improvement within this fixed benchmark set. The exploratory domain-composition resampling sensitivity crossed zero (−0.0213 to +0.0112), reflecting the limited number of independent held-out domains. Domain-adversarial training improved D-ECE1 in four domains and worsened it in three. The two domains with improved F1 under domain-adversarial training, neuroendocrine tumor and lung carcinoma, both had worse D-ECE1. The largest worsening was in lung carcinoma (+0.0373), whereas the largest improvements were in soft-tissue sarcoma (−0.0323) and cutaneous mast cell tumor (−0.0309). The Wilcoxon signed-rank *p* value was 0.8125 and the exact sign-test *p* value was 1.0; with seven paired domains, these tests are best read as descriptive rather than as proof of equivalence or absence of effect.

**Table 4:** Paired calibration change by held-out domain. Negative values favor domain-adversarial training.

Domain	Delta D-ECE1	95% paired case-bootstrap CI	Specimens
Breast carcinoma	−0.0006	−0.0134 to +0.0070	150
Neuroendocrine tumor	+0.0080	−0.0056 to +0.0096	55
Melanoma	−0.0143	−0.0278 to +0.0051	49
Lung carcinoma	+0.0373	+0.0290 to +0.0393	44
Lymphoma/lymphosarcoma	+0.0012	−0.0119 to +0.0127	55
Soft-tissue sarcoma	−0.0323	−0.0453 to −0.0219	100
Cutaneous mast cell tumor	−0.0309	−0.0396 to −0.0211	50
Mean paired delta	−0.0045	Fixed-domain: −0.0096 to −0.0014; exploratory domain-composition: −0.0213 to +0.0112	7 domains

The fixed-domain interval answers a narrow question conditioned on the observed seven domains and the seed-0 trained weights. It does not propagate domain-composition uncertainty beyond those seven domains or training-run variability beyond that seed. The seed-0 fixed-domain gain is therefore presented as a benchmark-specific observation rather than as a reproducible domain-adversarial calibration benefit.

### Maximum observed-domain calibration burden did not improve

The maximum observed-domain result was clearer than the macro average (Table 5). Canine lymphoma/lymphosarcoma had the highest observed D-ECE1 in both arms. Baseline maximum observed-domain D-ECE1 was 0.1804, with a raw bootstrap-maximum 95% interval from 0.1529 to 0.2118. Domain-adversarial maximum observed-domain D-ECE1 was 0.1816, with a raw bootstrap-maximum 95% interval from 0.1552 to 0.2105. The max-min spread was 0.1432 for baseline and 0.1450 for domain-adversarial training, and the cross-domain standard deviation was 0.0482 and 0.0495, respectively. Domain-adversarial training, therefore, did not reduce the maximum observed-domain calibration burden or the dispersion of calibration error across domains.

**Table 5:** Macro and maximum observed-domain calibration summaries.

Arm	Macro D-ECE1	Highest observed domain	Maximum observed D-ECE1	95% raw bootstrap-max CI	Max-min spread	SD
Baseline	0.0786	Lymphoma/lymphosarcoma	0.1804	0.1529–0.2118	0.1432	0.0482
Domain-adversarial	0.0741	Lymphoma/lymphosarcoma	0.1816	0.1552–0.2105	0.1450	0.0495

### Secondary seed sensitivity

The archived secondary seed-sensitivity series contained all 28 planned fold outputs: two additional training seeds, seven held-out domains, and two model arms. These runs were not mixed into the primary seed-0 final-protocol analysis. A post-hoc provenance audit of the two secondary seeds established their equivalence to the locked protocol: every one of the 28 runs completed all 20 planned training epochs; no non-finite optimizer step occurred in any run, so the recorded non-finite-step safeguard was never invoked (this safeguard field also varies within the verified primary series and is not protocol-defining); the training configuration was identical to the matched primary run on every setting except the intended seed; and the reported macro D-ECE1 changes reproduced exactly through the locked analysis path, with the primary seed-0 value recomputed as an in-audit control. The NPZ metadata retain a conservative provenance tag from generation time and were kept separate from the primary point estimate, but the audit confirms the secondary series is a valid protocol-sensitivity comparison rather than pilot output.

The macro D-ECE direction was not consistent across the two secondary seeds. In seed 1, baseline macro D-ECE1 was 0.0672 and domain-adversarial macro D-ECE1 was 0.0959, giving macro Delta D-ECE1 of +0.0287; domain-adversarial training improved D-ECE1 in four of seven domains but substantially worsened the highest observed-domain value, with canine lymphoma/lymphosarcoma reaching D-ECE1 0.2865. In seed 2, baseline macro D-ECE1 was 0.0647 and domain-adversarial macro D-ECE1 was 0.0579, giving macro Delta D-ECE1 of −0.0068; domain-adversarial training improved D-ECE1 in three of seven domains, and the highest observed domain changed from human neuroendocrine tumor in the baseline arm to canine soft-tissue sarcoma in the domain-adversarial arm.

The secondary values gave opposite macro Delta D-ECE1 directions relative to each other and to the seed-0 fixed-domain narrative (Fig. 1C). Across the three completed seed series, macro Delta D-ECE1 was −0.0045, +0.0287, and −0.0068. The between-series range was 0.0355, approximately eight times the seed-0 point estimate. The locked primary seed-0 analysis remains the source for the main point estimates, but its fixed-domain macro change should not be described as a reproducible domain-adversarial calibration benefit. Across the available series, maximum observed-domain D-ECE1 was not stably improved.

### Domain-adversarial training-log diagnostics

The saved domain-adversarial training logs provide a limited check on whether the adversarial branch was active. In the seven seed-0 domain-adversarial folds, the final recorded domain-head cross-entropy was close to the six-class chance cross-entropy (mean 1.8146, range 1.8121–1.8168; ln[6] = 1.7918), and the final gradient-reversal lambda was 0.9999. The same pattern appeared in the secondary seeds: mean final domain-head cross-entropy was 1.8140 for seed 1 and 1.8142 for seed 2, with final gradient-reversal lambda 0.9999 in each fold.

These logs support that the domain-adversarial branch was engaged under the intended schedule. They do not prove that the representation became domain invariant. The saved artifacts do not include domain-head accuracy, validation domain accuracy, or an independent frozen-feature source-domain probe, so the present study cannot distinguish a successfully confused domain head from a domain head whose loss was uninformative for other reasons.

### Count consequences at the fixed operating threshold

**Fig. 3:**
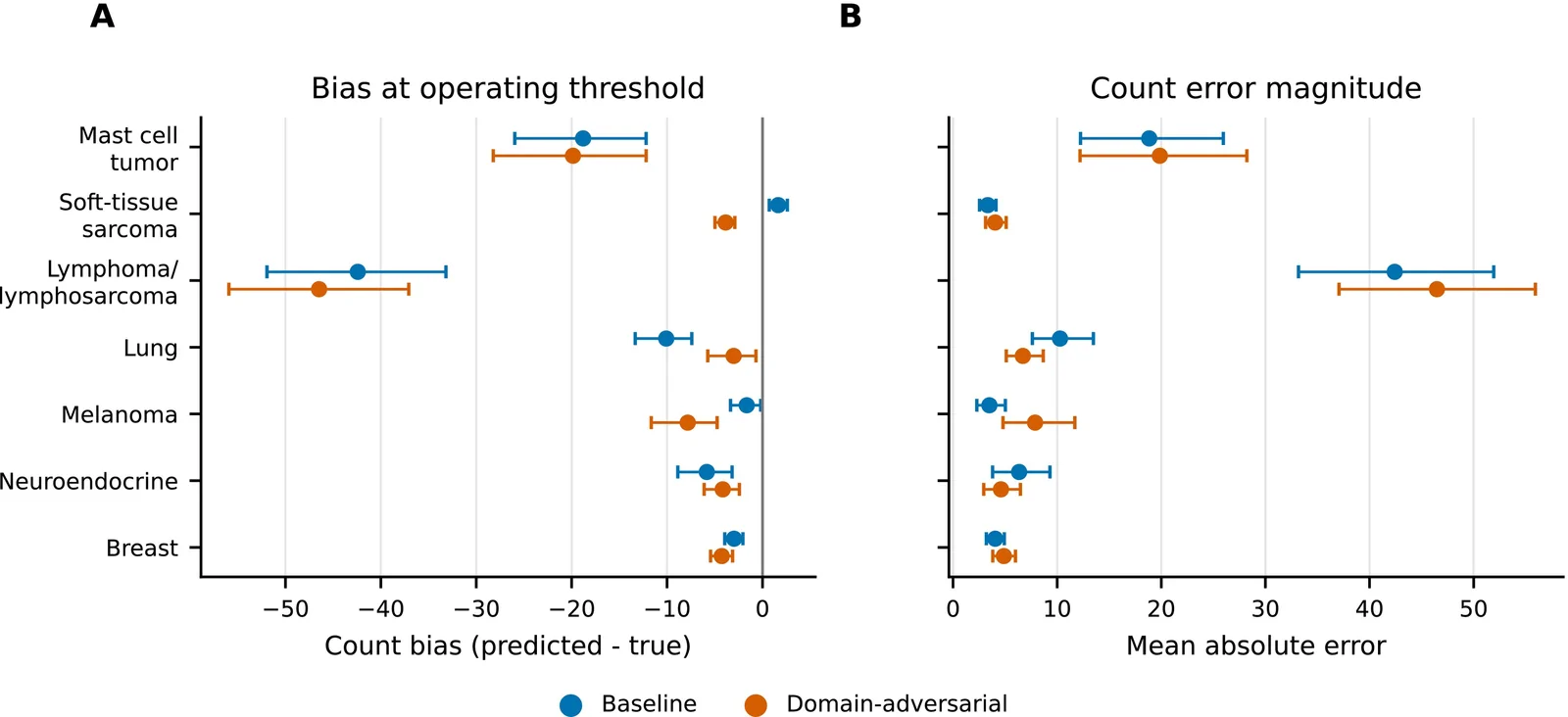
Case-level count consequences at the fixed operating threshold. Panel A shows count bias, defined as predicted minus true mitotic-figure count. Panel B shows count mean absolute error. Intervals are case-level bootstrap intervals.

At the operating threshold, count errors showed that detection reliability and count behavior are related but not interchangeable (Table 6, Fig. 3). Lymphoma/lymphosarcoma and cutaneous mast cell tumor had the largest raw count errors and strongest negative biases in both arms. In lymphoma/lymphosarcoma, baseline count MAE was 42.42 mitotic figures per specimen and domain-adversarial count MAE was 46.47; the corresponding predicted/true count ratios were 0.411 and 0.354. In cutaneous mast cell tumor, baseline count MAE was 18.84 and domain-adversarial count MAE was 19.86, with predicted/true ratios of 0.596 and 0.573. These values indicate marked undercounting at the fixed operating threshold in the domains with high mitotic-figure burden, whereas the normalized summaries distinguish burden from absolute count units.

**Table 6:** Case-level count consequences at the fixed operating threshold. Bias is predicted minus true count. Signed relative bias is the total bias divided by total true count; case-normalized absolute count error is the mean absolute case error divided by the case true count with a denominator floor of 1.

Domain	Arm	N	MAE	MAE 95% CI	Bias	Bias 95% CI	Pred/true	Signed rel. bias	Case-norm. error
Breast carcinoma	Baseline	150	4.05	3.19–4.93	−2.98	−3.95 to −2.05	0.740	−0.260	0.431
Breast carcinoma	Domain-adversarial	150	4.88	3.81–6.00	−4.27	−5.44 to −3.14	0.628	−0.372	0.445
Neuroendocrine tumor	Baseline	55	6.35	3.80–9.31	−5.84	−8.87 to −3.18	0.498	−0.502	0.656
Neuroendocrine tumor	Domain-adversarial	55	4.60	2.94–6.47	−4.16	−6.11 to −2.42	0.642	−0.358	0.475
Melanoma	Baseline	49	3.49	2.29–5.02	−1.65	−3.35 to −0.18	0.930	−0.070	0.347
Melanoma	Domain-adversarial	49	7.88	4.80–11.69	−7.84	−11.65 to −4.76	0.666	−0.334	0.357
Lung carcinoma	Baseline	44	10.27	7.61–13.48	−10.09	−13.34 to −7.41	0.481	−0.519	0.558
Lung carcinoma	Domain-adversarial	44	6.70	5.11–8.66	−3.02	−5.73 to −0.68	0.844	−0.156	0.556
Lymphoma/lymphosarcoma	Baseline	55	42.42	33.18–51.93	−42.42	−51.93 to −33.18	0.411	−0.589	0.550
Lymphoma/lymphosarcoma	Domain-adversarial	55	46.47	37.07–55.93	−46.47	−55.93 to −37.07	0.354	−0.646	0.616
Soft-tissue sarcoma	Baseline	100	3.33	2.54–4.13	+1.65	+0.71 to +2.61	1.128	+0.128	0.576
Soft-tissue sarcoma	Domain-adversarial	100	4.04	3.12–5.10	−3.86	−4.98 to −2.90	0.700	−0.300	0.365
Cutaneous mast cell tumor	Baseline	50	18.84	12.24–25.96	−18.80	−25.96 to −12.20	0.596	−0.404	0.492
Cutaneous mast cell tumor	Domain-adversarial	50	19.86	12.20–28.22	−19.86	−28.22 to −12.20	0.573	−0.427	0.425

The lymphoma/lymphosarcoma pattern was systematic rather than a mixture of overcounts and undercounts: MAE equaled the absolute value of the negative bias in both arms. With 3959 annotated mitotic figures across 55 specimens, the true mean burden was approximately 72 mitotic figures per specimen, whereas the fixed-threshold predictions undercounted by 42.42 and 46.47 mitotic figures per specimen in the baseline and domain-adversarial arms, respectively.

Domain-adversarial training improved count MAE in neuroendocrine tumor and lung carcinoma but worsened it in breast carcinoma, melanoma, lymphoma/lymphosarcoma, soft-tissue sarcoma, and cutaneous mast cell tumor. Lung carcinoma illustrates endpoint dissociation: F1 and count MAE improved, and the predicted/true ratio moved from 0.481 to 0.844, but D-ECE1 worsened by +0.0373, the largest calibration deterioration. Melanoma moved in the opposite direction: D-ECE1 improved by −0.0143, whereas F1 decreased and raw count MAE more than doubled; its predicted/true ratio moved from 0.930 to 0.666. This divergence is expected because D-ECE1 is detection-weighted among retained emissions, whereas count bias includes missed detections and is evaluated at a fixed operating threshold. These count summaries are not grade-threshold analyses. They quantify operating-point count consequences only; tumor-specific grade-threshold crossing risk was not reported because no cutoff was locked and citation-verified for this output.

### Inference-cap diagnostics and species regrouping

No inference window hit the detector's per-patch output cap in either arm, so the calibration tail was not truncated by the output limit in the final report. The confounded human/canine regrouping showed pooled human D-ECE1 of 0.053 for baseline and 0.047 for domain-adversarial training, and pooled canine D-ECE1 of 0.036 for baseline and 0.041 for domain-adversarial training. These are descriptive regroupings only. Because species is confounded with tumor type and scanner, these numbers do not estimate a species effect. The species-by-scanner design structure and the descriptive within-scanner calibration comparison are shown in Supplementary Fig. S1.

## Discussion

This benchmark shows why mitotic-figure detector reliability has to be measured directly. Across MIDOG++ LODO evaluation, operating-point F1, confidence calibration among retained emitted detections, count behavior, and the maximum observed-domain burden did not behave as interchangeable summaries. The domain-adversarial arm served as a controlled perturbation of the training objective: it produced a small average change in adaptive-bin D-ECE1 when the seven observed domains were treated as the fixed primary benchmark set, but that change was not robust to the exploratory domain-composition resampling sensitivity, was not directionally stable across the archived secondary seed series, and did not produce stable improvement in the maximum observed-domain D-ECE1. In short, this configuration did not establish a reproducible out-of-domain calibration benefit.

The domain pattern is the scientific payload. Lung carcinoma and neuroendocrine tumor had improved F1 under domain-adversarial training, yet both had worse D-ECE1. Lung carcinoma also had improved count MAE, showing that count behavior and emitted-detection calibration can diverge in the same held-out domain. Melanoma showed the converse: D-ECE1 improved, whereas F1 decreased and count MAE more than doubled. Soft-tissue sarcoma and cutaneous mast cell tumor had better D-ECE1 under domain-adversarial training despite lower F1. These discordant changes show that accuracy, calibration, and count consequences are not interchangeable endpoints. A method can show better operating-point detection and count behavior in one domain while increasing the confidence–precision gap among retained detections, or can improve confidence calibration while reducing operating-point F1.

The maximum observed-domain result is especially important. Canine lymphoma/lymphosarcoma had the highest observed D-ECE1 in both arms, with values around 0.18 and wide raw-bootstrap-maximum intervals. It also showed systematic undercounting at the fixed operating threshold: because MAE equaled the absolute negative bias in both arms, the average count error reflected near-uniform undercount rather than a cancellation of over- and undercounts. The MIDOG++ data descriptor identifies lymphosarcoma as a pronounced domain-shift setting and notes its smaller average cell size relative to other evaluated tumors.^1^ The present study did not determine which morphological, technical, or burden-related factors produced the calibration and count errors. Domain-adversarial training did not reduce the lymphoma/lymphosarcoma calibration burden and slightly widened the max-min spread. This matters because a fixed-domain macro average would make the domain-adversarial arm look slightly better, whereas the maximum observed-domain summary shows that the highest-error observed domain was essentially unchanged. This is a finite-benchmark disparity summary, not a guarantee about future domains.

The count summaries add a second operating-point caution. Lymphoma/lymphosarcoma and cutaneous mast cell tumor had large negative count biases at the fixed operating threshold in both arms. These are not grade-threshold results, and they should not be converted into claims about grade migration without tumor-specific cutoffs and threshold-specific matching. They show substantial undercounting in high-burden domains at this fixed threshold, even when F1 is reasonable in some domains. The predicted/true ratios and case-normalized absolute count error show why raw MAE alone is insufficient for comparing domains with different burdens. Calibration, F1, and count bias are related views of detector behavior, but none replaces the others.

The adversarial target itself is an important limitation. MIDOG++ domain identity is not a clean nuisance variable. It bundles tumor type, species, scanner, lab context, tissue architecture, cell density, mitotic burden, and mitotic morphology. Suppressing features predictive of that composite label could remove nuisance information, task-relevant pathology information, or both. The saved training logs show that the adversarial branch was active and ended with near-chance domain-head cross-entropy, but they do not contain domain-head accuracy or an independent frozen-feature probe. For that reason, the present result should be read as a test of this adversarial-training configuration against composite MIDOG++ domain labels, not as proof that domain-invariant representations were achieved or that adversarial training cannot improve calibration in better-factorial datasets.

The species-axis audit prevents an attractive but unsupported interpretation. MIDOG++ is valuable because it spans human and canine tumors, but it is not designed to identify a causal species effect. Human and canine domains do not share tumor types, and scanner context is nearly disjoint by species. The descriptive human/canine regrouping therefore cannot be interpreted as “the detector is better calibrated in one species.” The honest claim is design-based: MIDOG++ can support domain-level reliability measurement and can motivate comparative-pathology questions, but a species effect would require a dataset in which species crosses tumor type and scanner.

This study has several limitations. First, it compares two model arms within one architecture family and training budget. The result should not be generalized to all detectors, all domain-generalization methods, or all training schedules. Second, although a provenance audit confirmed the two additional seed series were generated under the locked protocol, three seeds still do not estimate the full distribution of training-run variability. Third, the maximum observed-domain D-ECE1 analysis has only seven domains. Case-level bootstrap intervals appropriately quantify uncertainty within held-out domains, but no resampling scheme creates more independent domains. The maximum observed-domain D-ECE1 should therefore be read as a finite-benchmark disparity summary, not as a high-powered between-domain hypothesis test or a guarantee about future domains. Fourth, D-ECE1 is detection-weighted among retained emitted detections and is silent about FNs; the count analysis was added precisely because confidence calibration among detections does not measure missed mitoses. Fifth, threshold-sweep F1 and grade-threshold crossing risk were not reported because the locked fold artifacts contain retained prediction coordinates and scores but not the corresponding ground-truth coordinates required for threshold-specific rematching, and no tumor-specific clinical cutoffs were locked. Sixth, the analysis did not add a separate image-quality score or post hoc slide-quality exclusion, so external validity remains conditional on the source dataset's selection and quality controls. Seventh, the annotations are point-centered, so matching-conditioned calibration is limited by the available ground-truth representation. Eighth, the matching radius is inherited from the official challenge metric; all results are conditional on that oracle.

The next experimental step is not only a larger model. More importantly, future benchmarks should save per-slide prediction coordinates and ground-truth coordinates in a form that permits threshold-specific rematching, grade-threshold analyses, and recalibration experiments without re-running inference. They should also include datasets in which species, tumor type, scanner, and lab context cross each other by design. Until then, maximum observed-domain calibration claims should stay close to the observed domains and should avoid causal species language.

In summary, this seven-domain MIDOG++ LODO audit shows that out-of-domain accuracy, confidence calibration among retained detections, count consequences, and maximum observed-domain D-ECE1 can move in different directions. Domain-adversarial training against composite MIDOG++ source-domain labels did not establish a reproducible calibration benefit in this controlled configuration, but its main value here was as a stress test revealing endpoint dissociation under domain shift. Mitotic-figure detector evaluation should therefore report detection-specific calibration, retained-detection denominators, count consequences, and finite-benchmark observed-domain D-ECE1 alongside F1, while respecting the identifiability limits of the dataset.

## Declaration of generative AI and AI-assisted technologies

During the preparation of this work, the author used OpenAI GPT-5.6 Pro for editorial assistance. After using this tool, the author reviewed and edited the content as needed and takes full responsibility for the content of the publication.

## CRediT authorship contribution statement

Cleverson de Souza is the sole author and was responsible for conceptualization, methodology, software, validation, formal analysis, investigation, data curation, writing – original draft, writing – review and editing, and visualization.

## Ethics statement

This study analyzed a public benchmark dataset and did not enroll new human participants or animals. The analysis relied on the published MIDOG++ benchmark and its associated ethical and data-use framework as described in the data descriptor.^1^

## Funding

No funding was received for this study.

## Declaration of competing interest

The author declares no competing interests.

## Data Availability

MIDOG++ is a public dataset described by Aubreville et al.^1^ A reproducibility release has been prepared with the analysis code, run manifest, final prediction-output NPZ files, final JSON outputs, generated report artifacts, manuscript tables, publication figures, QC records, archive metadata templates, and checksums. The release records the vendored evalutils 0.3.1 matching/scoring code used for the official MIDOG centroid-distance assignment. The release supports full numerical recomputation of all reported metrics, figures, and tables from the deposited prediction-output NPZ files and JSON outputs. Reproducing inference or de novo training additionally requires the raw MIDOG++ images and annotations, which are not redistributed in that bundle; readers should obtain them through the official public dataset route and comply with the dataset terms. The trained model checkpoints are not included in the release; they are documented by SHA-256 checksum in the release manifest and can be deposited separately on request. The reproducibility release is archived on Zenodo at https://doi.org/10.5281/zenodo.21329254 under a CC-BY-4.0 license.

## Appendix A Supplementary data

Supplementary Fig. S1 Structural identifiability of the species axis in MIDOG++. Panel A shows the species-by-scanner contingency structure, emphasizing that tumor type does not cross species and scanner is nearly disjoint by species. Panel B shows the descriptive 3DHistech canine tumor window; these values do not estimate a species effect.

## References

[1] Aubreville M., Wilm F., Stathonikos N. (2023). A comprehensive multi-domain dataset for mitotic figure detection. Sci Data.

[2] Aubreville M., Stathonikos N., Donovan T.A. (2024). Domain generalization across tumor types, laboratories, and species: insights from the 2022 edition of the Mitosis Domain Generalization Challenge. Med Image Anal.

[3] Aubreville M., Stathonikos N., Bertram C.A. (2023). Mitosis domain generalization in histopathology images: the MIDOG challenge. Med Image Anal.

[4] Guo C., Pleiss G., Sun Y., Weinberger K.Q. (2017). Proceedings of the 34th International Conference on Machine Learning.

[5] Kuppers F., Kronenberger J., Shantia A., Haselhoff A. (2020). Proceedings of the IEEE/CVF Conference on Computer Vision and Pattern Recognition Workshops.

[6] Kuppers F., Haselhoff A., Kronenberger J., Schneider J. (2022). Deep Neural Networks and Data for Automated Driving.

[7] Chen R.J., Wang J.J., Williamson D.F.K. (2023). Algorithmic fairness in artificial intelligence for medicine and healthcare. Nat Biomed Eng.

[8] Sagawa S., Koh P.W., Hashimoto T.B., Liang P. (2020). Proceedings of the 8th International Conference on Learning Representations.

[9] Ganin Y., Ustinova E., Ajakan H. (2016). Domain-adversarial training of neural networks. J Mach Learn Res.

[10] Jahanifar M., Raza M., Xu K. (2025). Domain generalization in computational pathology: survey and guidelines. ACM Comput Surv.

[11] Nixon J., Dusenberry M.W., Zhang L., Jerfel G., Tran D. (2019). Proceedings of the IEEE/CVF Conference on Computer Vision and Pattern Recognition Workshops.

[12] Naeini M.P., Cooper G.F., Hauskrecht M. (2015). Obtaining well calibrated probabilities using Bayesian binning. Proc AAAI Conf Artif Intel.

[13] Lin T.-Y., Goyal P., Girshick R., He K., Dollar P. (2017). Proceedings of the IEEE International Conference on Computer Vision.

