# Supplementary material for: Metric discordance under MIDOG++ domain shift: a leave-one-domain-out audit of F1, emitted-detection confidence calibration, and ROI count error
Source: J Pathol Inform. 2026 Jul 31;22:100705. doi: 10.1016/j.jpi.2026.100705 (PMC13524644; doi:10.1016/j.jpi.2026.100705)
Supplement: Supplementary file 1 [file mmc1.pdf]

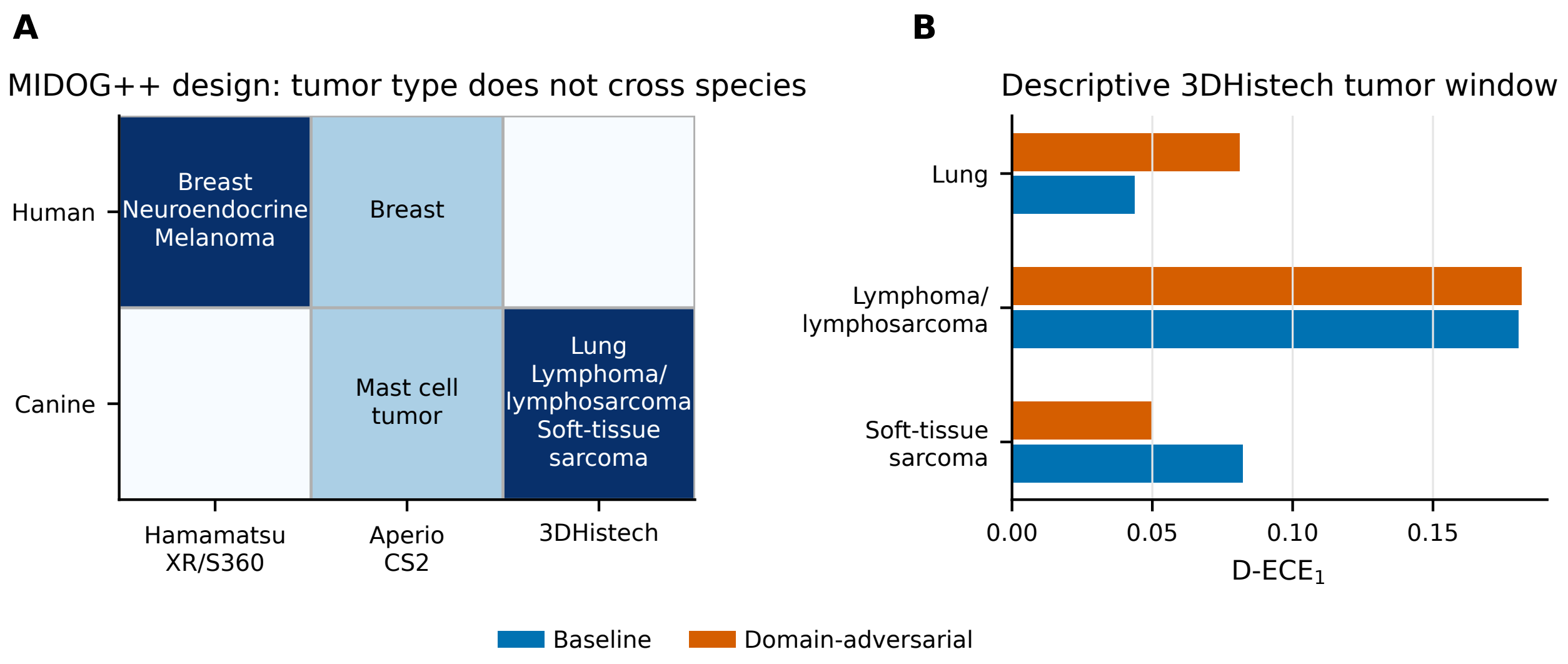
A
MIDOG++ design: tumor type does not cross species
Human
Canine
Breast
Neuroendocrine
Melanoma
Breast
Mast cell
tumor
Lung
Lymphoma/
lymphosarcoma
Soft-tissue
sarcoma
Hamamatsu
XR/S360
Aperio
CS2
3DHistech
B
Descriptive 3DHistech tumor window
Lung
Lymphoma/
lymphosarcoma
Soft-tissue
sarcoma
0.00
0.05
0.10
0.15
D-ECE$_1$
Baseline
Domain-adversarial
